# C-955-07. Identification and Functional Profiling of Potential Prognostic miRNA Biomarkers for Inhalation Injury Presence and/or Severity

**DOI:** 10.1093/jbcr/irag033.156

**Published:** 2026-04-06

**Authors:** Tarryn Kay Prinsloo, Wayne George Kleintjes, Kareemah Najaar

**Affiliations:** Cape Peninsula University of Technology, Cape Town, Western Cape; Sheik Khalifa Hospital Fujairah, Cape Town, Western Cape; Cape Peninsula University of Technology, Cape Town, Western Cape

## Abstract

**Introduction:**

Inhalation injury contributes significantly to burn morbidity and mortality yet no diagnostic consensus exists and treatment remains largely supportive. Current diagnostic tools could be strengthened by incorporating biomarkers that are patient- and disease-specific, robust, and easily detectable. Circulating microRNAs (miRNAs) possess these attributes and have emerged as potential indicators of injury response and outcomes. We hypothesized that distinct differentially expressed (DE) miRNA signatures would correlate with the presence and/or severity of inhalation injury, and regulate related pathological pathways.

**Methods:**

Blood samples (n = 59) were collected on or shortly after admission from burn patients treated at a specialized burn centre for adults, over an 18-month period. Total RNA was extracted, with quality and quantity assessed. miRNA profiling was performed on exemplar samples (mild and severe inhalation injury cases) using high-throughput sequencing. Differential expression analysis was conducted with EdgeR in R and corroborated by DESeq2. Fisher’s Exact test compared DE miRNAs between groups. Overlapping DE miRNAs meeting adjusted *p*<.05 and fold change >1.5 were subjected to downstream analysis: target gene prediction (miRNet), protein–protein interaction networks (STRING), top 10 hub genes (cytoHubba), and functionally enriched pathway (EnrichR platforms: GO, KEGG, Reactome, PANTHER).

**Results:**

Ten DE miRNAs overlapped and met significance thresholds. Nine were notably up-regulated in severe injury (miR-143-3p, miR-200b-3p, miR-148b-5p, miR-10b-5p, miR-30a-5p, miR-15a-5p, miR-374a-5p, miR-21-5p, miR-144-5p) and one was down-regulated in mild injury (miR-504-5p). Target gene and network analysis revealed key hub genes that were predominantly enriched in inflammatory and apoptotic signaling pathways.

**Conclusions:**

This study identifies a potential panel of distinct DE miRNAs associated with inhalation injury severity, underscoring their regulatory role in inflammation and apoptosis, as supported by both literature and the observed pathway enrichment. These preliminary findings provide a foundation for developing circulating miRNA biomarkers to aid in the prognosis of inhalation injury presence and severity in burn patients.

**Applicability of Research to Practice:**

The identification of circulating miRNA signatures offers a minimally invasive approach for prognostic evaluation of inhalation injury. Incorporating such biomarkers into clinical practice could enable earlier risk stratification, guide treatment intensity, and improve outcome prediction in burn patients. In the longer term, pathway insights may inform the development of targeted therapies addressing the molecular drivers of injury severity.

**Funding for the Study:**

N/A.

